# Designed Syntheses of Three {Ni_6_PW_9_}-Based Polyoxometalates, from Isolated Cluster to Cluster-Organic Helical Chain

**DOI:** 10.3390/molecules27134295

**Published:** 2022-07-04

**Authors:** Chong-An Chen, Yan Liu, Guo-Yu Yang

**Affiliations:** MOE Key Laboratory of Cluster Science, School of Chemistry and Chemical Engineering, Beijing Institute of Technology, Beijing 102488, China; cca@bit.edu.cn (C.-A.C.); liuyanik@163.com (Y.L.)

**Keywords:** polyoxometalates, hydrothermal syntheses, cluster-organic frameworks, helical chain

## Abstract

Three new hexa-Ni-substituted Keggin-type polyoxometalates (POMs), [Ni_6_(OH)_3_- (DACH)_3_(H_2_O)_6_(PW_9_O_34_)]·31H_2_O (**1**), [Ni(DACH)_2_][Ni_6_(OH)_3_(DACH)_3_(HMIP)_2_(H_2_O)_2_(PW_9_O_34_)]·56 H_2_O (**2**), and [Ni(DACH)_2_][Ni_6_(OH)_3_(DACH)_2_(AP)(H_2_O)_5_(PW_9_O_34_)]·2H_2_O (**3**) (DACH = 1,2-Diami- nocyclohexane, MIP = 5-Methylisophthalate, AP = Adipate) were successfully made in the presence of DACH under hydrothermal conditions. **1** is an isolated hexa-Ni-substituted Keggin unit decorated by DACH. In order to further construct POM cluster-organic frameworks (POMCOFs) on the basis of **1**, by analyzing the steric hindrances and orientations of the POM units, the rigid HMIP and flexible AP ligands were successively incorporated, and another anionic monomeric POM **2** and the new 1D POM cluster organic chain (POMCOC) **3** were obtained. HMIP ligand still acts as a decorating group on the Ni_6_ core of **2** but results in the different spatial arrangement of the {Ni_6_PW_9_} units. AP ligands in **3** successfully bridge adjacent isolated POM cluster units to 1D POMCOC with left-hand helices. The AP in **3** is the longest aliphatic carboxylic acid ligand in POMs, and the 1D POM cluster-AP helical chain represents the first 1D POMCOC with a helical feature.

## 1. Introduction

In the past century, polyoxometalates (POMs) have been widely researched for their abundant structures and applications in catalytic [1,2,3], magnetic [4], and electrical fields [5,6]. In order to enrich POMs’ structural chemistry and further expand or optimize their applications, researchers have started to design and construct POM cluster organic frameworks (POMCOFs) [7,8,9] which is a new and promising branch of cluster organic frameworks (COFs) [10,11,12]. Since the POMCOF was reported [13], considerable efforts have been made in building POMCOFs with Keggin-/Anderson-/Lindqvist-POM secondary building units (SBUs) and rigid aromatic organic linkers [7,8,14,15]. However, compared with the traditional MOFs, the designed syntheses of POMCOFs are still facing huge challenges for the following two reasons: (1) POM clusters have large negative charges and oxygen-rich surfaces, which facilitate their bonding to metal cations, rather than the O-/N-donors from organic linkers. (2) POMs are rigid and stable clusters, therefore, the steric hindrance effects of POM SBUs and linkers need to be well-matched during assembly. Hence, how to choose proper POM SBUs and organic linkers is the key to constructing POMCOFs.

Among seven typical types of POMs, only Anderson-/Lindqvist-/Keggin-types have been successfully applied as SBUs in POMCOFs. Since 2016, the first Anderson-type POM-based heterometallic cluster organic framework was made; Anderson-type POMs have become the popular choice for SBUs [8]. The combination of Anderson-type SBU and rigid bifunctional tris(alkoxo) ligand with a pyridyl group opens up the gate of Anderson-type POMCOFs’ world. Lindqvist-type POMs are important members of the POMs family. Though five different elements can all produce the Lindqvist-type [M_6_O_19_]^n−^ (M = V^V^, Nb^V^, Ta^V^, Mo^VI^, W^VI^) cluster, only polyoxovanadates have been successfully applied as SBUs in Lindqvist-type POMCOFs [15,16]. So far, most of the reported POMCOFs are made with Keggin-type POM SBUs [7,13,14,17,18,19,20]. In these POMCOFs, most of the SBUs are saturated {ε-M_4_PMo_12_O_40_} (M=La, Zn) [13,14,17,18,19], of which, the incorporation of M (M=Zn^2+^, La^3+^) provide the easier bonding sites than the saturated {PMo_12_} units for organic linkers. Our group has long been devoted to transition metal substituted POMs (TMSPs) based on the trilacunary Keggin fragments under hydrothermal conditions. From our perspective, the trilacunary sites of the [XW_9_O_34_] (P, W, Ge) unit can act as structure-directing agents (SDAs) to induce transition metal ions’ aggregation to cluster, on which the terminal end of water molecules may facilitate the substitutions of organic linkers in constructing Keggin-type POMCOFs. Since the first hexa-Ni^II^ substituted TMSP based on trilacunary Keggin fragments was made [4], we have been working on POMs structural chemistry based on hexa-Ni^II^-substituted POMs and have already mastered the synthetic conditions of hexa-Ni^II^ substituted Keggin POMs. By using {Ni_6_PW_9_} SBUs and rigid aromatic carboxylate ligands, we have built a series of novel Keggin-type POMCOFs [7,20]. Hence, we believe that some other intriguing POMCOFs can be made by using {Ni_6_PW_9_} SBUs with proper organic linkers.

Rigid and semi-rigid aromatic carboxylate ligands are the common linkers being used in making POMCOFs [7,20,21,22]; their rigid structures are favorable for the stabilization of the frameworks. However, the large steric hindrance effects of POMs and rigid ligands sometimes cannot match to form POMCOFs. To overcome this difficulty, alipha- tic dicarboxylic acid may be a potential candidate due to its smaller steric hindrances and better flexibilities, which may produce some intriguing frameworks with helical or interpenetrating features that cannot be obtained with rigid aromatic ligands. However, little relevant research has been made, including two typical examples containing aliphatic dicarboxylic acid-bridges for a 2D POMCOF and a tetramer [23,24]. Hence, in this work, we first made an isolated hexa-Ni-substituted Keggin-type POM [Ni_6_(OH)_3_- (DACH)_3_(H_2_O)_6_(PW_9_O_34_)]·31H_2_O (**1**) under hydrothermal conditions. The abundant terminal water molecules on the Ni_6_ cores are potential substitution sites for organic linkers, which help us to further construct POMCOFs. When we first applied the rigid carboxylate ligand MIP, another hexa-Ni-substituted Keggin-type monomer [Ni(DACH)_2_]- [Ni_6_(OH)_3_(DACH)_3_(HMIP)_2_-(H_2_O)_2_(PW_9_O_34_)]·56H_2_O (**2**) was obtained, HMIP ligands still decorate on the Ni_6_ cores, failing to bridge the POM clusters. By analyzing the structure of **2**, we used the aliphatic dicarboxylate AP ligands as a linker and a new 1D POMCOC with helical chains [Ni(DACH)_2_][Ni_6_(OH)_3_(DACH)_2_(AP)(H_2_O)_5_(PW_9_O_34_)]·2H_2_O (**3**) was made. To the best of our knowledge, the AP in **3** is the longest aliphatic dicarboxylic acid being incorporated in POMs. Moreover, the 1D helical chain of **3** is the first 1D POMCOC with helical features.

## 2. Experimental Section

### 2.1. General Procedure

All the reagents were analytical grade and used without any further purification. Na_10_[A-α-PW_9_O_34_]·7H_2_O was prepared by a method from the literature [25]. Meso-form DACH was used in the syntheses. The powder X-Ray diffraction (PXRD) patterns of the three compounds were collected on a Bruker D8 Advance X-ray diffractometer (Bruker, Karlsruhe, Germany) with Cu Kα radiation (λ = 1.54056 Å) and 2θ scanning from 5–50°. UV-Vis absorption spectra were obtained on a Shimadzu UV3600 spectrometer (Shimadzu, Kyoto, Japan) with wavelengths from 190 to 800 nm. IR spectra were recorded on a Nicolet iS10 FT-IR spectrometer (Thermo Fisher Scientific, Waltham, MA, USA) with the wavenumbers ranging from 4000 to 400 cm^−1^. Thermogravimetric analyses were conducted on a Mettler Toledo TGA/DSC 1100 analyzer (Mettler Toledo, Zurich, Switzerland) heating up from 25–1000 °C (heating rate: 10 °C/h) under an air atmosphere. Elemental analyses proceeded on the EuroEA3000 elemental analyzer (EuroVector, Pavia, Italy).

### 2.2. Syntheses

#### 2.2.1. Synthesis of **1**

**Scheme 1:**
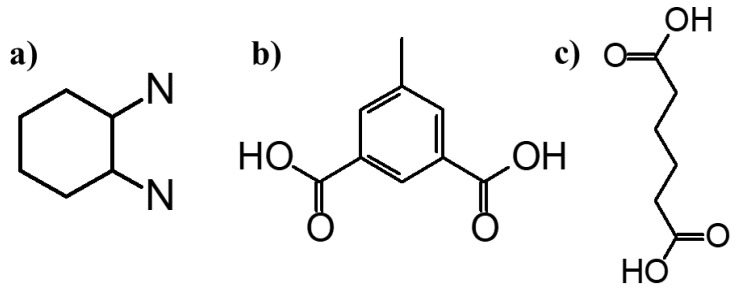
DACH (**a**), MIPA (**b**), and AA (**c**) ligands in **1**–**3**.

Na_9_[A-α-PW_9_O_34_]·7H_2_O (0.320 g, 0.125 mmol) and NiCl_2_·6H_2_O (0.820 g, 3.44 mmol) were stirred in 9 mL 0.5 mol/L sodium acetate buffer (pH = 4.8) for 10 min; then, 3 mL DACH (Scheme 1a) was slowly dropped in and continually stirred for 30 min. The resulting solution was sealed in a 25 mL Teflon-lined stainless-steel autoclave and heated at 170 °C for 5 days. After cooling down to room temperature and washing with distilled water, green rod-like crystals were obtained with a yield of 34% (based on Na_9_[A-α- PW_9_O_34_]·7H_2_O). Elemental analysis calcd (%): C, 5.93; H, 3.26; and N, 2.30 (based on [Ni_6_(OH)_3_(DACH)_3_(H_2_O)_6_(PW_9_O_34_)] -·31H_2_O). Found: C, 7.10; H, 2.17; N, 2.81. IR(KBr, cm^–1^): 3428(s), 3332(w), 3280(w), 2929(w), 2856(w), 1628(w), 1588(w), 1449(w), 1377(w), 1231(w), 1119(w), 1039(s), 941(vs), 842(vs), 796(vs), and 716(vs).

#### 2.2.2. Syntheses of **2** and **3**

The synthetic procedures of **2** and **3** were the same as **1,** except for the adding of MIPA (5-Methylisophthalic Acid, Scheme 1b) (0.200 g, 1.11 mol) and AA (Adipic Acid, Scheme 1c) ligands (0.200 g, 1.37 mol) for **2** and **3**, with the yield of 36% and 28%, respectively. Based on Na_9_[A-α-PW_9_O_34_]·7H_2_O. Elemental analysis calcd (%) for **2**: C, 12.34; H, 4.35; and N, 3.00 (based on [Ni(DACH)_2_][Ni_6_(OH)_3_(DACH)_3_(HMIP)_2_(H_2_O)_2_(PW_9_O_34_)]·- 56 H_2_O). Found: C, 14.10; H, 3.37; N, 3.39. IR(KBr, cm^–1^): 3458(s), 3338(w), 3264(w), 2929(w), 2859(w), 1562(w), 1361(w), 1033(w), 935(vs), 841(vs), 796(vs), and 715(vs). Elemental analysis calcd (%) for **3**: C, 10.53; H, 2.36; and N, 3.27 (based on [Ni(DACH)_2_][Ni_6_(OH)_3_- (DACH)_2_(AP)(H_2_O)_5_(PW_9_O_34_)]·2H_2_O). Found: C, 10.44; H, 2.63; and N, 3.34. IR(KBr, cm^–1^): 3423(s), 3240(w), 2923(w), 2860(w), 1591(w), 1546(w), 1413(w), 1037(s), 943(vs), 848(vs), 796(vs), and 710(vs).

### 2.3. X-ray Crystallography

The single-crystal diffraction data of **1**–**3** were collected on a Gemini A Ultra CCD diffractometer with graphite monochromated Mo Kα (Λ = 0.71073 Å) radiation at 296(2) K. The structures were solved by direct methods and refined by the full-matrix least-squares fitting on F^2^ method with the SHELX-2008 program package [26]. Anisotropic displacement parameters were refined for all atomic sites except for some disordered atoms. The contribution of the disordered solvent molecules in **1** and **2** was treated with the SQUEEZE method in PLATON (Utrecht University, Utrecht, The Netherlands). In the refinements, 0, 1, and 2 lattice water molecules were found for **1**–**3** from the Fourier maps, respectively. Based on the potential solvent-accessible voids and electron counts from the SQUEEZE reports, there were 31 and 55 lattice water molecules removed for **1** and **2**, respectively. According to the elemental analyses and TGA, there are 27 and 34 lattice water molecules lost from efflorescence in **1** and **2**, respectively. In **3**, 4 absorbed water molecules were found. Basic crystallographic data and structural refinement data are listed in Table 1. Detailed crystallographic data have been deposited on the Cambridge Crystallographic Data Centre: CCDC 2171028 (for **1**), 2170965 (for **2**), and 2170966 (for **3**). These data can be obtained free of charge via http://www.ccdc.cam.ac.uk/conts/retrieving.html accessed on 29 June 2022 or from the Cambridge Crystallographic Data Centre, 12 Union Road, Cambridge CB2 1EZ, UK; Fax: +44-1223-336-033; or email: deposit@ccdc.cam.ac.uk.

## 3. Result and Discussion

### 3.1. Structure of **1** and Designed Syntheses for **2**

**Figure 1:**
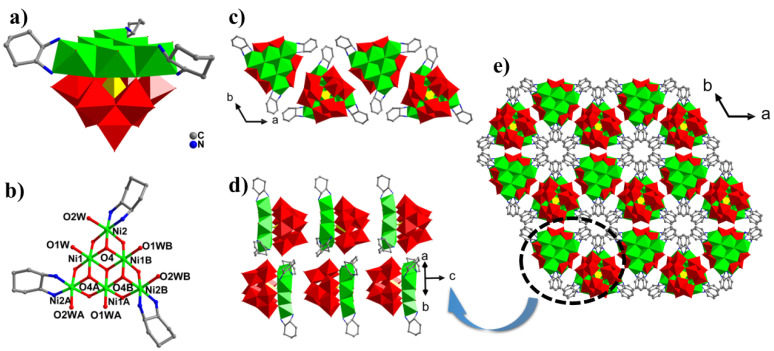
(**a**) A polyhedral view of polyoxoanion **1a**.; (**b**) View of the Ni_6_ cluster in **1a**; (**c**,**d**) Shoulder-to-shoulder arrangements of **1a** with opposite orientations along the *a*-axis and [110] direction; and (**e**) The 3D supramolecular framework of **1**. Color code of polyhedral: WO_6_: red; NiO_6_/NiO_4_N_2_: green; and PO_4_: yellow. Hydrogen atoms of the ligands are not shown for better clarity.

X-ray diffraction analyses reveal that **1** crystallizes in the trigonal space group *P*-3*c*1, consisting of the neutral [Ni_6_(*μ*_3_-OH)_3_(DACH)_3_(H_2_O)_6_(PW_9_O_34_)] (**1a**, Figure 1a) cluster. **1a** can be seen as the classical trilacunary Keggin [B-α-PW_9_O_34_]^9−^ fragment being capped by a triangular [Ni_6_(*μ*_3_-OH)_3_]^9+^ cluster. Due to the trigonal *C*_3_ symmetry of **1**, there are only two independent Ni^2+^ in the Ni_6_ cluster (Figure S1, Supplementary Materials). Each Ni1 and Ni2 interconnect with each other by edge-sharing, producing three edge-sharing {Ni_3_O_4_} truncated cubanes. Three Ni1O_6_ octahedra locate on the three lacunary sites of the {PW_9_} unit, while three Ni2O_4_N_2_ octahedra are on the three vertexes of the triangular Ni_6_ cluster, further decorated by three DACH ligands, respectively (Figure 1b). According to BVS calculations [27], the bond valance of *μ*_3_-O4 is 1.12, indicating its protonation. **1a** exhibits two opposite orientations, which are alternately arranged with a shoulder-to-shoulder arrangement along the *a*-axis and [110] direction (Figure 1c,d). Such arrangements construct the snowflake-like supramolecular channels with *S_6_* symmetry and are the hydrophobic voids as well (Figure 1e).

The presence of six terminal water molecules on the Ni_6_ cluster provides abundant substituted sites for organic ligands. We started to incorporate organic ligands into the reaction system of **1**, attempting to construct POMCOFs with proper organic linkers. In our previous work, we have successfully made two 1D POMCOCs {[Ni_6_(OH)_3_(H_2_O)_2_- (enMe)_3_(PW_9_O_34_)](1,3-bdc)}[Ni(enMe)_2_]·4H_2_O (**4,** enMe = 1,2-diaminopropane, 1,3-bdc = 1,3-benzenedicarboxylate acid) and {[Ni_6_(OH)_3_(H_2_O)(en)_4_(PW_9_O_34_)](Htda)}·H_3_O·4H_2_O (**5,** en = ethylenediamine, tda = thiodiglycolic acid) based on {Ni_6_PW_9_} SBUs and V-type rigid dicarboxylate ligands (1,3-bdc and tda) [7]. To analyze these structures carefully, we found that in **4** and **5**, {Ni_6_PW_9_} SBUs are arranged in shoulder-to-shoulder and face-to-face modes, respectively, which are further bridged by the V-type dicarboxylate ligands to 1D POMCOCs. In **1**, though the opposite-orientated {Ni_6_PW_9_} units exhibit shoulder-to-shoulder arrangements along the *a*-axis, the interunit distances are too close to accommodate the organic ligands. Hence, we choose the similar V-type ligand MIP to see if the methyl group can further spread out the opposite orientated POM units and if the carboxyl groups can bridge adjacent same orientated units to 1D chains at the same time. By adding MIPA into the reaction of **1**, **2** was obtained. The observation of **2** confirms part of our speculations; though HMIP still acts as a decoration group, it changes the orientations of adjacent POM units such that two different orientated units both arrange in shoulder-to-shoulder modes separately with moderate interunit distances.

### 3.2. Structure of **2** and Designed Syntheses for **3**

**Figure 2:**
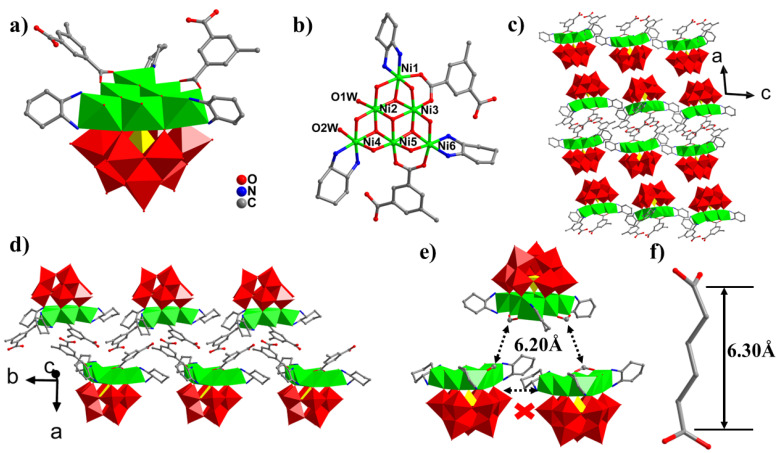
(**a**) A polyhedral view of polyoxoanion **2a**.; (**b**) View of the Ni_6_ cluster in **2a**; (**c**) Spatial arrangements of **2a** along with the *a-*and *c-* axes; (**d**) Spatial arrangements of **2a** along the *b*-axis; (**e**) Interunit distances of **2a**; and (**f**) Matchable distance of AP ligand. Color code of polyhedral: WO_6_: red; NiO_6_/NiO_4_N_2_: green; and PO_4_: yellow. Hydrogen atoms of the ligands are not shown for better clarity.

X-ray diffraction analyses reveal that **2** crystalizes in its monoclinic space group *P*2_1_/c. Its polyoxoanionic cluster [Ni_6_(OH)_3_(DACH)_3_(HMIP)_2_(H_2_O)_2_(PW_9_O_34_)] (**2a**) is similar to that of **1a**, except for four water molecules in **1a** being replaced by two HMIP ligands in **2a** (Figure 2a,b and Figure S1). This difference makes **2a** an anionic cluster, accompanied by the charge-balancing [Ni(DACH)_2_]^2+^ complex, in which Ni^2+^ exhibit the planar square coordination geometry (Figure S2, Supplementary Materials).

Due to the large steric hindrance of HMIP, adjacent opposite-orientated POM clusters are spread out and adopt face-to-face arrangements with each other, while the same orientated units still maintain shoulder-to-shoulder arrangements (Figure 2c,d), which are ideal arrangements for making POMCOFs based on our previous research [7,20]. Using another organic linker with a longer length may help to achieve our aims, but the longer length corresponds to the larger steric hindrances, which may affect the orientations of POM units or increase the interunit distances. Rigid aromatic carboxylic ligands seem unlikely to satisfy our design. Hence, we transfer our focus to chainlike aliphatic dicarboxylic acids. Their higher flexibilities may facilitate their bridging functions on POM SBUs with more flexible orientations and interunit distances and may further result in some intriguing interpenetrating or helical structures that cannot be obtained with rigid aromatic carboxyl ligands. We found that the bilateral DACH molecules on each Ni_6_ cluster prevent the bridging of adjacent same-orientated SBUs with organic linkers (Figure 2d,e). Additionally, the distance between two terminal –COOH groups from adjacent opposite-orientated POM SBUs is 6.20 Å (Figure 2e), which is nearly matchable with that of AP in the reported polymers (6.30 Å, Figure 2f) [28]. Using AP to replace HMIP ligand in **2** may achieve our goals. Based on the above considerations, AP was used as a linker in the synthesis of **3**. Under similar synthetic conditions with **1** and **2**, **3** was obtained. AP ligand successfully bridges adjacent opposite orientated POM cluster units to the unpreceded 1D helical chains.

### 3.3. Structure of **3**

**Figure 3:**
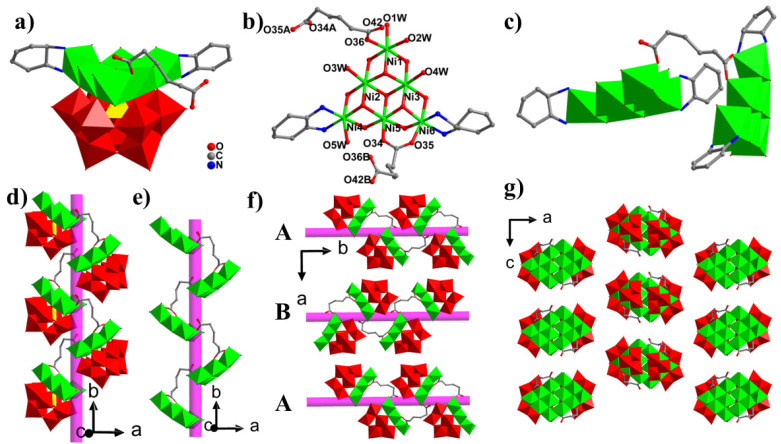
(**a**) A polyhedral view of polyoxoanion **3a**.; (**b**) View of the Ni_6_ cluster and its’ linkage with AP; (**c**) The linkage of AP with adjacent Ni_6_ cluster; (**d**) View of the 1D helical chains with left-hand helices along the *b*-axis; (**e**) The simplified sketch of the Ni_6_-AP 1D chain; and (**f**,**g**) The -ABAB- and -AAA- stacking modes along *a*-and and *c*-axis, respectively. Color code of polyhedral: WO_6_: red; NiO_6_/NiO_4_N_2_: green; and PO_4_: yellow. Hydrogen atoms of the ligands are not shown for better clarity.

**3** crystallizes in the orthorhombic space group *P*2_1_2_1_2_1_. Its asymmetric unit contains a [Ni_6_(OH)_3_(DACH)_2_(AP)(H_2_O)_5_(PW_9_O_34_)] (**3a**) cluster (Figure 3a), a [Ni(DACH)_2_]^2+^ complex, and two lattice water molecules (Figure S1, Supplementary Materials). Compared with **1a** and **2a**, only four-terminal water molecules are substituted by two bidentate DACH ligands on the Ni_6_ cluster of **3a** (Figure 3b). Each Ni_6_ cluster links with two AP, of which, one terminal carboxyl group of the AP replaces two terminal water molecules on Ni5 and Ni6, while another carboxyl group replaces only one water molecule on Ni1 (Figure 3b). Each AP ligand bridges two Ni_6_ clusters (Figure 3c). Such substitution and linkage successfully construct the 1D helical chain with left-hand helices around a 2_1_-screw axis (Figure 3d,e). Adjacent 1D chains stack in -ABAB- and -AAA- sequences along the *a-*and *c*-axis, respectively (Figure 3f,g). It is worth noting that the orientation of each POM SBU and interunit distance have been continually adjusted to the face-to-shoulder arrangements with shorter interunit distance to match the linkage of AP ligand, which are different from those in rigid dicarboxylate ligand-bridged POMCOFs. Such special arrangements of POM SBUs, and the good flexibility of AP, synergistically contribute to the 1D helical chains with left-hand helices. Similar to that in **2**, [Ni(DACH)_2_]^2+^ complexes with planar square configuration locate interchain to compensate for the negative charges of the chains (Figure S2, Supplementary Materials).

### 3.4. Structural Comparisons

**Figure 4:**
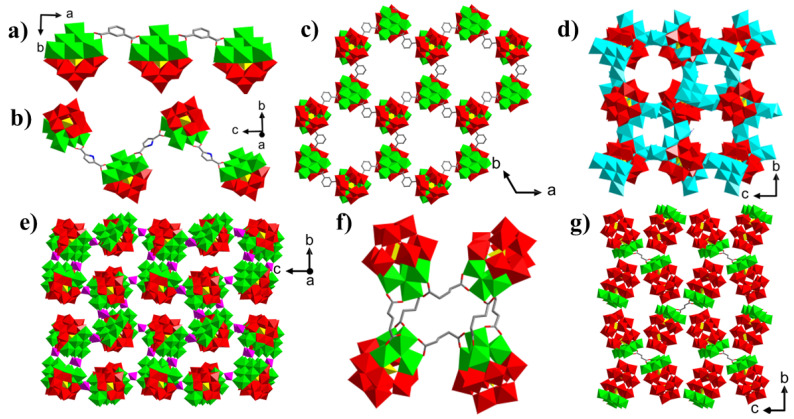
(**a**) One-dimensional chain built from {Ni_6_PW_9_} unit and 1,3-bdc ligand; (**b**) One- dimensional chain built from {Ni_6_PW_9_} unit and tda ligand; (**c**) Two-dimensional layer built from {Ni_6_PW_9_} unit and 1,3-bdc ligand; (**d**) Three-dimensional framework built from the interconnection of {Cu_6_PW_9_} unit through Cu–O=W linkage; (**e**) Three-dimensional framework built from {Ni_6_PW_9_} unit and WO_4_ tetrahedron; (**f**) Tetramer built from {Ni_4_SiW_9_} and glutaric acid linker; and (**g**) Two-dimensional layer built from Dawson-type {Ni_6_P_2_W_15_} unit and succinic acid linker. Color code of polyhedral: WO_6_: red; NiO_6_/NiO_4_N_2_: green; PO_4_: yellow; CuO_6_: light blue; and WO_4_: purple. Hydrogen atoms of the ligands are not shown for better clarity.

In TMSPs’ abundant structural chemistry, POM clusters have various linkages with each other to generate different 1D/2D/3D structures:

First, the interconnections of POM clusters (including different structural types) and rigid aromatic organic ligands. This linkage produces most of the 3D POMCOFs, while 1D chains and 2D layers are relatively rare through this connection, except for these three examples: the 1D chains built from the {Ni_6_PW_9_} unit and 1,3-bdc, tda ligand (Figure 4a,b) [7], respectively, and the layer made by another ethylenediamine-func- tionalized {Ni_6_PW_9_} unit and 1,3-bdc ligand (Figure 4c) [20].

Second, the interconnections of TMSP cluster units through TM-O=W bonds. This linkage generates a series of 1D chains and 2D layers [29,30]. The 3D open frameworks constructed by the pure TM–O=W linkage are only observed in Cu^II-^substituted TMSPs, including [{Cu_6_(μ_3_-OH)_3_(en)_3_(H_2_O)_3_}(B-α-PW_9_O_34_)]·7H_2_O and [Cu_6_(μ_3_-OH)_3_(en)_3_(H_2_O)_3_(B- α-PW_9_O_34_)]·4H_2_O (Figure 4d), which are caused by the unique Jahn–Teller effect of CuO_4_N_2_ octahedra with the axial elongation [31,32].

Third, the TMSP frameworks with TM complex-bridges. TM complex-bridges are common in TMSPs’ frameworks. They can extend the POM units to 1D/2D/3D frameworks through TM–O=W, TM–O–TM, and TM–N⋯N-TM linkages [33,34,35,36].

Fourth, is the TMSP framework with WO_4_ bridges. However, to the best of our knowledge, it was only found in the first chiral 3D framework of [Ni(enMe)_2_]_3_[WO_4_]_3_- [Ni_6_(enMe)_3_(OH)_3_PW_9_O_34_]_2_·9H_2_O (Figure 4e) [37].

Compared with these TMSP-based frameworks with four different linkages, the 1D helical chains in **3** represent a new structural type of POMCOCs. Aliphatic dicarboxylic acid ligands are rare not only in POMCOFs but also in POMs. Limited evidence includes the glutaric acid-bridged tetramer [{(SiW_9_O_34_)Ni_4_(OH)_3_}_4_(OOC(CH_2_)_3_COO)_6_] (Figure 4f) and the succinic acid-bridge hexa-substituted Dawson-type-based layer [Ni_6_(μ_3_-OH)_3_- (dap)_2_(en)(H_2_O){OOC(CH_2_)_2_COO}_0.5_(CH_3_COO)(P_2_W_15_O_56_)] (Figure 4g) [23,24]. The AP in **3** is the longest aliphatic dicarboxylic acid being incorporated in POMs family. Moreover, it differs from those 1D chains with a TM–O=W linkage and 1D POMCOCs featuring strict chains [7,29]; the 1D helical chains in **3** are the first 1D POMCOC with helical features.

Since the hexa-Ni^II^ cluster of **1**–**3** is similar to those in the reported hexa-Ni- substituted TMSPs, we compared their bond lengths and bond angles to speculate the magnitude properties of the title compounds. As shown in Table S1 (Supplementary Materials), the Ni–O bond lengths and Ni–O–Ni bond angles of **1**–**3** are in the ranges of 1.915–2.295 Å and 90.9–114.2°, respectively. According to the previous research [4,7,31,38,39], when the Ni–O–Ni bond angles are in the range of 90–104°, ferromagnetic exchange interactions are dominant. When Ni–O–Ni bond angles are larger than 104°, anti-ferromagnetic exchange interactions may exist. When ferromagnetic and antiferromagnetic behaviors coexist, the overall magnetic behaviors are determined by which one is dominant. Normally, most of the Ni–O–Ni bond angles in the hexa-Ni^II^ cluster are in the ferromagnetic dominant ranges when ferromagnetic and antiferromagnetic coupling coexistences appear. Ferromagnetic exchange behaviors are expected for hexa-Ni^II^ clusters, which have been proved by the measurements in our previous research [4,7,31,38,39]. In **1**, since all the Ni–O–Ni bond angles are in the range of 92.5–102.1°, ferromagnetic exchange behaviors are expected. In **2** and **3**, the Ni–O–Ni bond angles are in the range of 90.9–106.8° and 91.6–114.2°, respectively, indicating the coexistences of ferromagnetic and antiferromagnetic couplings. There are only 1 and 2 Ni–O–Ni bond angles larger than 104°, indicating that the ferromagnetic exchange behaviors are dominant in **2** and **3**, similar to those reported in hexa-Ni^II^-substituted TMSPs.

### 3.5. Powder XRD Patterns

As shown in Figure S3 (Supplementary Materials), the experimental PXRD patterns of **1**–**3** were all consistent with the simulated patterns obtained from single-crystal data, which confirm the purities of the samples. The differences in the intensities were attributable to the preferred orientations.

### 3.6. IR Spectra

As shown in Figure S4 (Supplementary Materials), the IR spectra of **1**–**3** show a series of similar absorption bands ranging from 4000–400 cm^−1^. The wide absorption bands from 3528 to 3134 cm^−1^ are assigned to the stretching vibrations of the –OH, –CH_2,_ and –NH_2_ groups. The sharp absorption peaks from 3005 to 2817 cm^−1^ are the stretching vibrations of the –CH_2_ and –NH_2_ groups. The peaks ranging from 1652–1363 cm^−1^ are the characteristic peaks of the bending vibrations of –NH_2_ and –CH_2_ groups in **1**–**3** and the carboxylate groups of the carboxylate ligands in **2**–**3**. Four intense characteristic peaks of *v*(P–O), *v*(W–O_t_), *v*(W–O_b_), and *v*(W–O_c_) of the Keggin-fragments are observed at 1039, 941, 842, 796, and 716 cm^−1^ for **1**, 1033, 935, 841, 796, and 715 cm^−1^ for **2**, and 1037, 943, 848, 796, and 710 cm^−1^ for **3**, respectively.

### 3.7. UV-Vis Absorption Spectra

In order to investigate the optical properties of the title compounds, UV-Vis absorption and optical diffuse reflectance spectra of **1**–**3** were obtained in the wavelength range of 190–800 nm. As shown in Figure S5 (Supplementary Materials), the optical band gaps of **1**–**3** are 2.60, 2.59, and 2.56 eV, respectively, which are comparable to other Ni_6_-substitute POMs, including [Ni_6_(μ_3_-OH)_3_(en)_2_(dien)(H_2_O)_5_(B-α-PW_9_O_34_)]·3H_2_O (2.42 eV), [Ni_6_(μ_3_-OH)_3_(dap)_2_(py)_6_- (H_2_O)(B-α-PW_9_O_34_)]·H_2_O (2.37 eV), and [Ni(en)_2_][Ni_6_(μ_3_-OH)_3_(en)_3_(1,3-bdc)(H_2_O)_2_(B-α- PW_9_O_34_)]·9H_2_O (2.53 eV) [20,29]. It was found that the band gaps of **1–3** are in the order of **3** < **2** < **1**, which conforms to the band gaps of the compounds decreasing with the increasing dimensionality or complexity of the structures, as proposed by Kanatzidis and Papavassiliou [40].

## 4. Conclusions

In summary, three new TMSPs containing {Ni_6_PW_9_} units were designed and synthesized from monomers to 1D POMCOC under hydrothermal conditions. **1** is a monomer with DACH molecules decorating the Ni_6_ cluster. In order to construct POMCOF on the basis of **1**, the rigid aromatic MIP ligand was first incorporated and the anionic monomeric POM **2** was obtained. HMIP still acts as a decorating group on the Ni_6_ cluster but fails to bridge adjacent {Ni_6_PW_9_} units. By analyzing the orientations and steric hindrance between adjacent {Ni_6_PW_9_} units of **2**, the aliphatic AP ligand was purposely chosen to replace HMIP on the base of **2**, which resulted in the formation of **3**, a new 1D POMCOC with novel helical chain. Owing to the good flexibility of the AP linker, **3** represents the first 1D POMCOC with a helical chain. This work is an example of our continued work of constructing POMCOFs with hexa-Ni^II^ substituted TMSP SBUs. The successfully designed syntheses from **1** to **3** provide us with a new strategy of using chainlike dicarboxylate acid as a linker to make POMCOFs, which may lead to some intriguing structures that cannot be found with rigid aromatic linkers. Further works with this strategy are in progress.

## Figures and Tables

**Table 1:** Crystallographic data and structural refinements for **1**–**3**.

	1	2	3
Formula	Ni_6_PW_9_O_74_C_18_H_119_N_6_	Ni_7_PW_9_O_103_C_48_H_203_N_10_	Ni_7_PW_9_O_48_C_30_H_81_N_8_
Molecular weight	3642.06	4665.78	3418.61
Crystal system	Trigonal	Monoclinic	Orthorhombic
Space group	*P*-3*c*1	*P*2_1_/*c*	*P*2_1_2_1_2_1_
*a*/Å	18.1775	24.9850	36.6105
*b*/Å	18.1775	14.9500	14.1605
*c*/Å	21.3261	26.6410	13.8492
*α*/°	90	90	90
*β*/°	90	98.697	90
*γ*/°	120	90	90
*V*/Å^3^	6102.5	9837	7179.7
*Z*	4	4	4
*Dc*/g cm^−3^	3.964	3.151	3.163
*μ*/mm^−1^	18.880	11.956	16.263
*F*(000)	6840	9048	6312
Goodness-of-fit on *F*^2^	1.075	1.099	1.131
*R* indices [*I* > 2*σ*(*I*)] ^1^	0.0355 (0.0946)	0.0633 (0.1624)	0.0601 (0.1350)
*R* indices (all data)	0.0504 (0.1043)	0.0996 (0.1777)	0.0704 (0.1413)

^1^ *R*_1_ = Σ||*F*_0_| − |*F*_c_||/Σ|*F*_0_|. *wR*_2_ = {Σw[(*F*_0_)^2^ − (*F*_c_)^2^]^2^/Σw[(*F*_0_)^2^]^2^}^1/2^.

## Data Availability

Not applicable.

## Supplementary Materials

The following supporting information can be downloaded at: https://www.mdpi.com/article/10.3390/molecules27134295/s1, Table S1: Comparisons of the bond lengths and bond angles in Ni_6_-subsi- tuted TMSPs; Figure S1: Asymmetric units of **1**–**3**; Figure S2: [Ni(DACH)_2_]^2+^ complex in **2** and **3**; Figure S3: PXRD of **1**–**3**. Figure S4: IR spectra of **1**–**3**; Figure S5: UV-Vis spectra of **1**–**3**; Figure S6: TG curves of **1**–**3**. [41,42,43] are cited in the Supplementary Materials.

## Sample Availability

Samples of the compounds are available from the authors.
