# Supplementary material for: Designed Syntheses of Three {Ni6PW9}-Based Polyoxometalates, from Isolated Cluster to Cluster-Organic Helical Chain
Source: Molecules. 2022 Jul 4;27(13):4295. doi: 10.3390/molecules27134295 (PMC9268654; doi:10.3390/molecules27134295)
Supplement: Supplementary file 1 [file molecules-27-04295-s001.zip › molecules-1763917-supplementary.pdf]

molecules

Supplementary Materials

Designed Syntheses of Three {Ni_6PW_9}-Based Polyoxometalates, from Isolated Cluster to Cluster-Organic Helical Chain

Chong-An Chen, Yan Liu and Guo-Yu Yang *

MOE Key Laboratory of Cluster Science, School of Chemistry and Chemical Engineering, Beijing Institute of Technology, Beijing 102488, China; cca@bit.edu.cn (C.-A.C.); liuyanik@163.com (Y.L.)
* Correspondence: ygy@bit.edu.cn or ygy@fjirsm.ac.cn

Table S1. Comparisons of the bond lengths and bond angles in Ni_6-subsituted TMSPs.
Figure S1. Asymmetric units of **1–3**.
Figure S2. $[Ni(DACH)_2]^{2+}$ complex in **2** and **3**.
Figure S3. PXRD of **1–3**.
Figure S4. IR spectra of **1–3**.
Figure S5. UV-Vis spectra of **1–3**.
Figure S6. TG curves of **1–3**.

Table S1. Comparisons of the bond lengths and bond angles in Ni_6-subsituted TMSPs.

Ni_6-subsititued TMSPs	Ni–O distance	Ni–O–Ni bond angles	Numbers of the Ni–O–Ni bond angles (90-104°)	Numbers of the Ni–O–Ni bond angles (>104°)	ref
$Ni_6(\mu_3\text{-}OH)_3(H_2O)_6(enMe)_3(B\text{-}\alpha\text{-}SiW_9O_{34})$	2.011–2.158 Å	90–104°	18	0	4
$Ni_6(\mu_3\text{-}OH)_3(H_2O)_2(enMe)_3(B\text{-}\alpha\text{-}SiW_9O_{34})$(1,3-bdc)	1.980–2.286 Å	91.0–104.4°	17	1	7
$Ni_6(\mu_3\text{-}OH)_3(H_2O)_6(dap)_3(B\text{-}\alpha\text{-}SiW_9O_{34})$	1.990–2.171 Å	91.7–105.2°	17	1	31
$Ni_6(Tris)(en)_3(BTC)_{1.5}(B\text{-}\alpha\text{-}PW_9O_{34})$	1.989–2.224 Å	90.8–103.6°	18	0	38
$Ni_6(\mu_3\text{-}OH)_3(H_2O)_4(OAc)(B\text{-}\alpha\text{-}SiW_9O_{34})$	1.980–2.293 Å	90.7–107.3°	17	1	39
$Ni_6(\mu_3\text{-}OH)_3(DACH)_3(H_2O)_6(B\text{-}\alpha\text{-}PW_9O_{34})$ (**1**)	1.992–2.175 Å	92.5–102.1°	18	0	This work
$Ni_6(\mu_3\text{-}OH)_3(DACH)_3(H_2O)_2(HMIP)_2(B\text{-}\alpha\text{-}PW_9O_{34})$ (**2**)	1.991–2.295 Å	90.9–106.8°	17	1	
$Ni_6(\mu_3\text{-}OH)_3(DACH)_2(H_2O)_5(AP)_2(B\text{-}\alpha\text{-}PW_9O_{34})$ (**3**)	1.915–2.261 Å	91.6–114.2°	16	2	

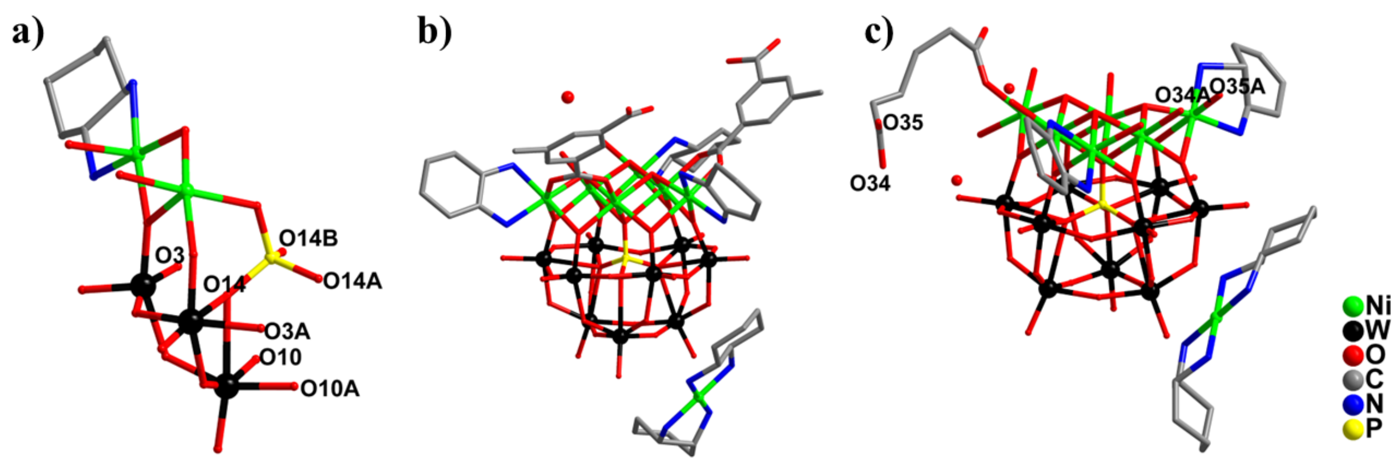

Figure S1. Asymmetric units of **1**–**3**.

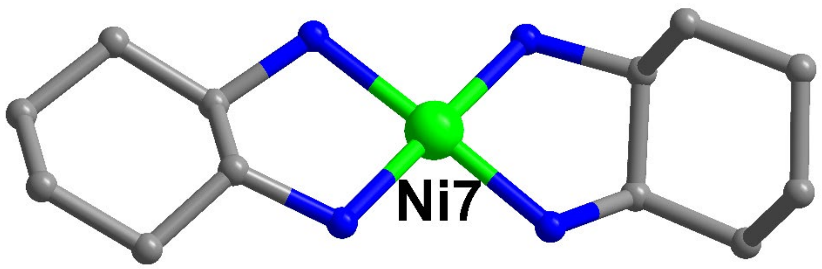

Figure S2. $[Ni(DACH)_2]^{2+}$ complex in **2** and **3**.

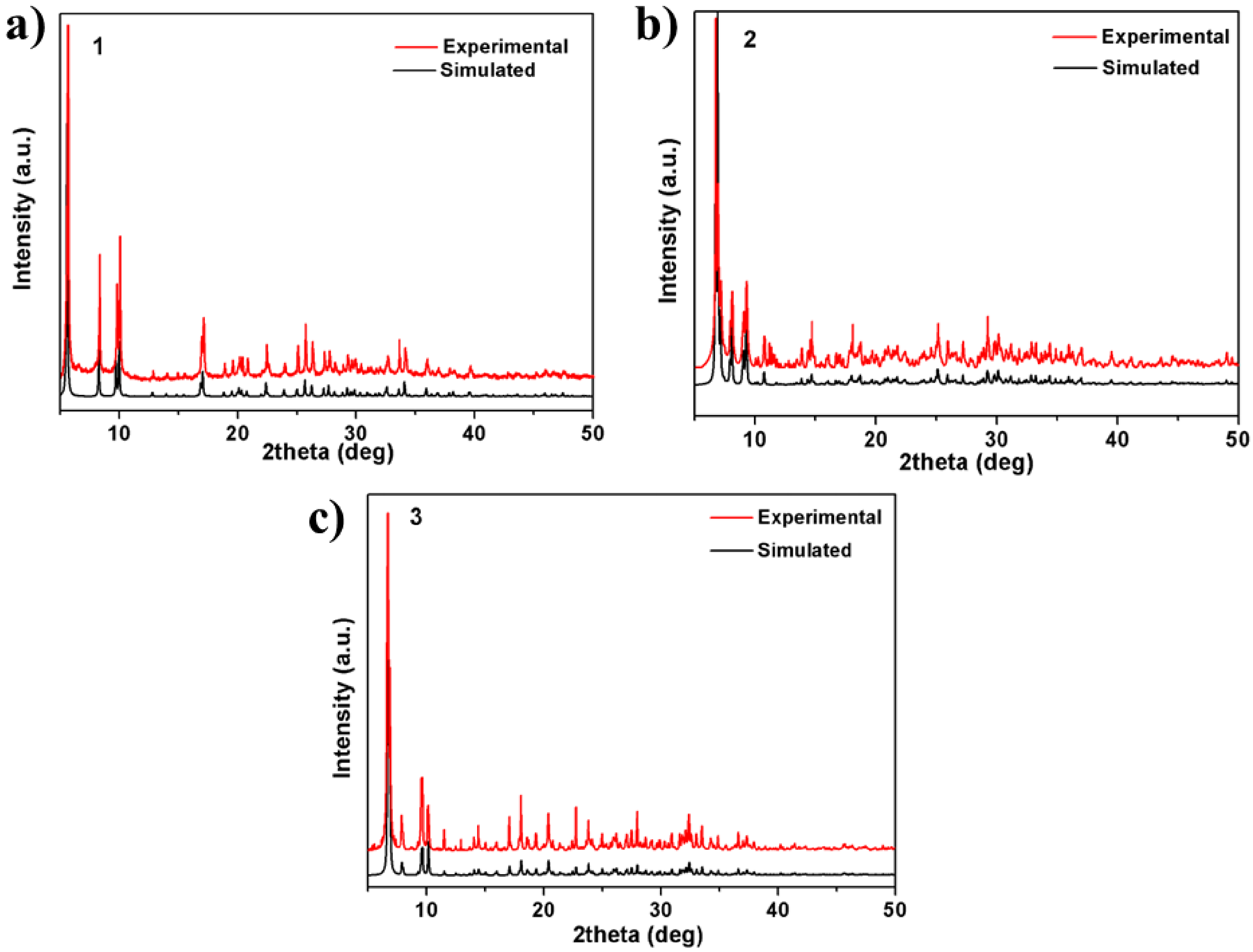

Figure S3. PXRD of **1**–**3**.

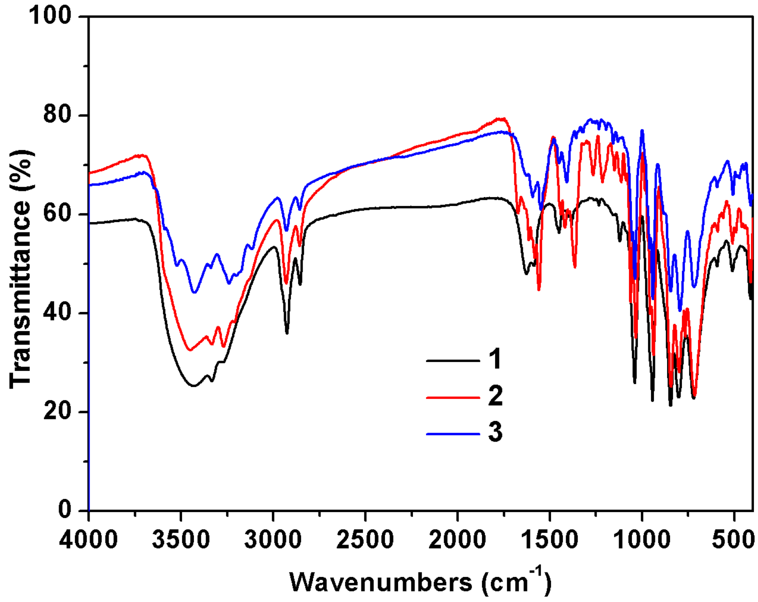

Figure S4. IR spectra of **1**–**3**.

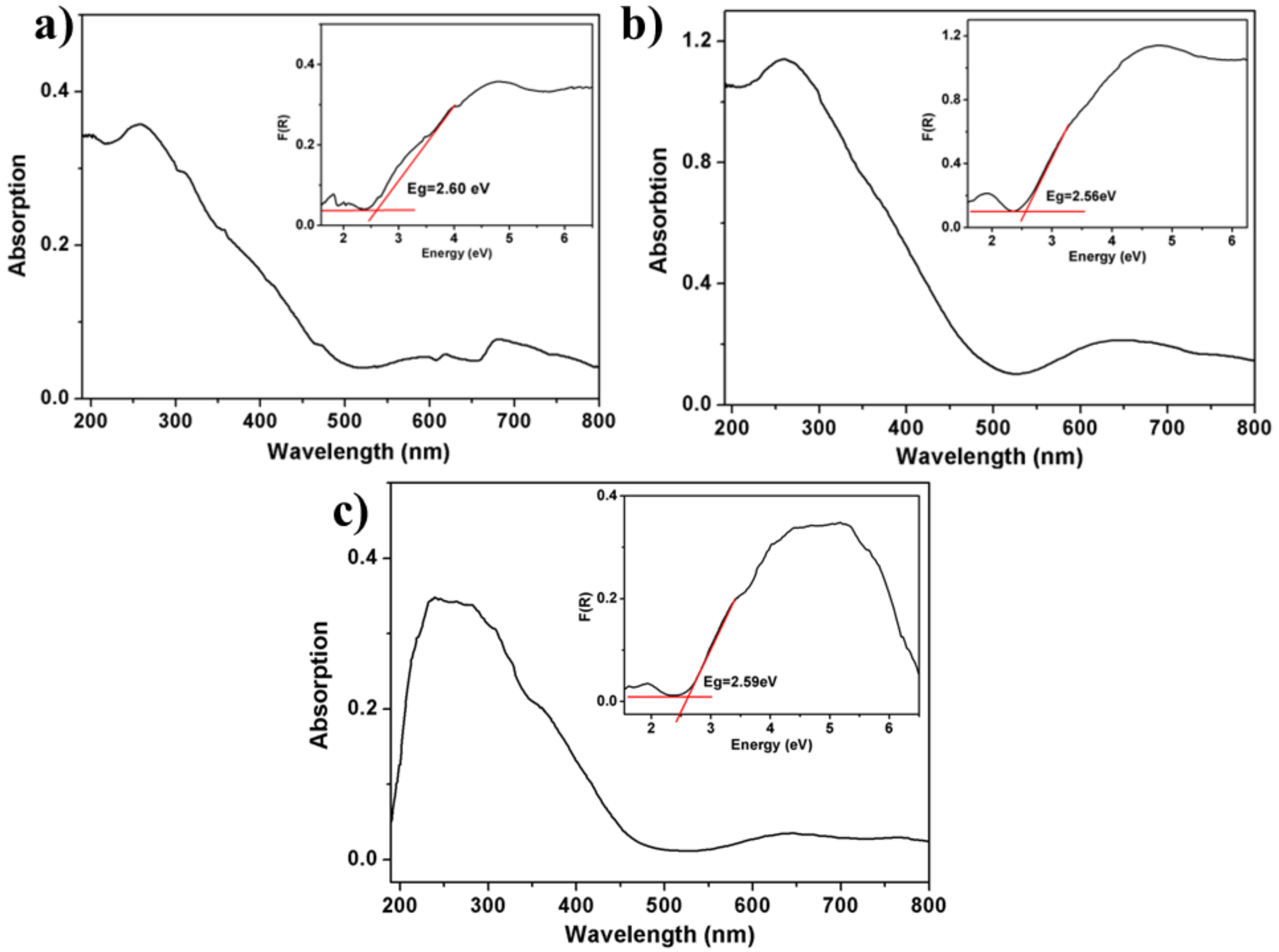

Figure S5. UV-Vis spectra of **1**–**3**.

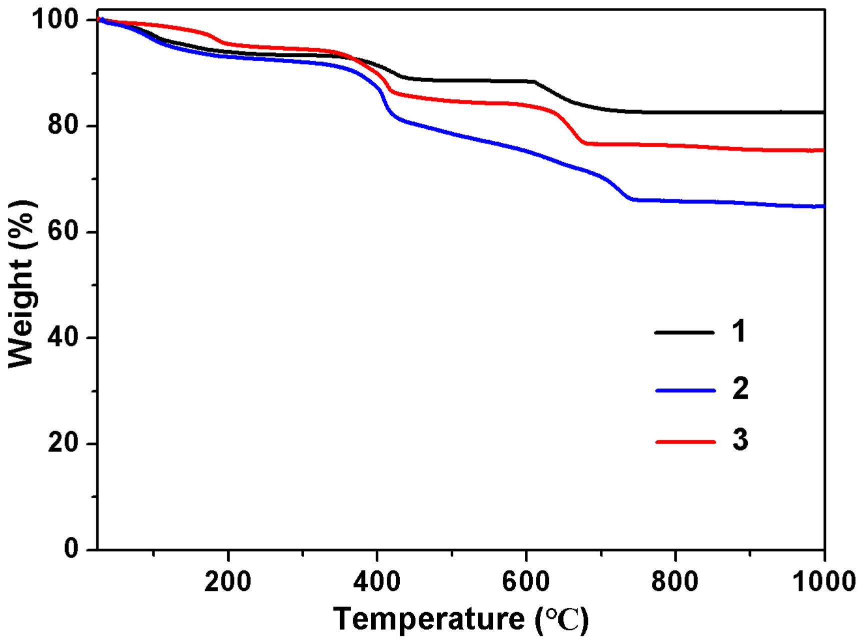

Figure S6. TG curves of **1**–**3**.

The thermal stabilities of **1–3** were measured from 25–1000 ℃ with the heating rate of 10 ℃/min under the air atmosphere. The TG curves of **1–3** show that their weight losses are all conducted by multistep (Figure S6).

According to the elemental analyses, the inconsistences between calculated and experimental values of **1** and **2** indicate that there exists partial weight loss after X-ray analyses and before TG/elemental analyses. This part of the weight loss can be attributed to the loss of lattice water molecules since these water molecules are not stable in POMs, especially in those lattice water-rich POMs, which are not unusual in POMs [41–43]. The total numbers of the lost lattice water molecules are first calculated from elemental analyses (27 water molecules for **1**, 34 water molecules for **2**) and further confirmed by TGA. The analyses of TG curves of **1–3** are as follow:

For **1**, The total observed weight loss of 17.57% are in consistence with the calculated value of 17.39% (based on $[Ni_6(OH)_3(DACH)_3(H_2O)_6(PW_9O_{34})]\cdot 4H_2O$). The first weight loss of 5.85% (Cal: 5.70%) from 25–259 ℃ are assigned to the removal of 4 lattice water molecules and 6 coordinated water molecules. The continually weight loss of 11.72% (Cal: 11.69%) from 355–741 ℃ are attributed to the loss of 3 DACH molecules and 1.5 water molecules corresponding to the dehydrations of 3 OH groups.

For **2**, the total weight loss of 34.58% are in agreement with the calculated value (33.81%, based on $[Ni(DACH)_2]$-$[Ni_6(OH)_3(DACH)_3(HMIP)_2(H_2O)_2(PW_9O_{34})]\cdot 22H_2O$). The first 10.39% (Cal: 10.65%) weight loss from 25-335 ℃ correspond to the removal of 22 lattice water molecules and 2 coordinated water molecules, while the second stage weight loss of 24.19% (Cal: 23.16%) from 335-841 ℃ are the release of 5 DACH molecules, 2 MIPA ligands (2 HMIP and 2 H from 2 OH groups) and 0.5 water molecules from the dehydrations of 1 OH groups.

For **3**, there are three steps for the 24.24% (Cal: 22.07%, based on $[Ni(DACH)_2][Ni_6(OH)_3(DACH)_2(AP)(H_2O)_5$-$(PW_9O_{34})]\cdot 2H_2O$) weight loss from 74-748 ℃. The first step of 5.51% weight loss is more than the calculated value (3.68%) from 74-240 ℃, indicating that except for the release of 2 lattice water molecules and 5 coordinated water molecules, there exists 4 absorbed water molecules on the surfaces of the samples. The second step of 10.66% (Cal: 10.94%) weight loss from 348-546 ℃ are assigned to 1 AA ligands (1 AP and 2 H from 2 OH groups) and 2 DACH molecules from the Ni-complex. The third step weight loss of 8.07% (Cal: 6.93%) from 582-748 ℃ are the removal of the remaining 2 DACH molecules on the Ni_6 cluster and 0.5 water molecules (1 OH group).
